# Quantitative Profiling of Long-Chain Bases by Mass Tagging and Parallel Reaction Monitoring

**DOI:** 10.1371/journal.pone.0144817

**Published:** 2015-12-11

**Authors:** Christer S. Ejsing, Mesut Bilgin, Andreu Fabregat

**Affiliations:** Department of Biochemistry and Molecular Biology, VILLUM Center for Bioanalytical Sciences, University of Southern Denmark, Odense, Denmark; Université PARIS- DIDEROT (7), FRANCE

## Abstract

Long-chain bases (LCBs) are both intermediates in sphingolipid metabolism and potent signaling molecules that control cellular processes. To understand how regulation of sphingolipid metabolism and levels of individual LCB species impinge upon physiological and pathophysiological processes requires sensitive and specific assays for monitoring these molecules. Here we describe a shotgun lipidomics method for quantitative profiling of LCB molecules. The method employs a “mass-tag” strategy where LCBs are chemically derivatized with deuterated methyliodide (CD_3_I) to produce trimethylated derivatives having a positively charged quaternary amine group. This chemical derivatization minimizes unwanted in-source fragmentation of LCB analytes and prompts a characteristic trimethylaminium fragment ion that enables sensitive and quantitative profiling of LCB molecules by parallel reaction monitoring on a hybrid quadrupole time-of-flight mass spectrometer. Notably, the strategy provides, for the first time, a routine for monitoring endogenous 3-ketosphinganine molecules and distinguishing them from more abundant isomeric sphingosine molecules. To demonstrate the efficacy of the methodology we report an in-depth characterization of the LCB composition of yeast mutants with defective sphingolipid metabolism and the absolute levels of LCBs in mammalian cells. The strategy is generic, applicable to other types of mass spectrometers and can readily be applied as an additional routine in workflows for global lipidome quantification and for functional studies of sphingolipid metabolism.

## Introduction

Long-chain bases (LCBs) are low abundant biosynthetic intermediates in sphingolipid metabolism and also signaling molecules that impinge on a wide range of physiological processes [1,2]. The first step in *de novo* sphingolipid metabolism is the condensation of serine and fatty acyl-CoAs to produce the 3-ketosphinganines. This reaction is catalyzed by serine palmitoyltransferase, which in yeast and mammalian cells primarily uses palmitoyl-CoA to produce C_18_-3-ketosphingosine. Serine palmitoyltransferase can also utilize other fatty acyl-CoAs to produce LCBs with different hydrocarbon chain lengths and numbers of double bonds. 3-ketosphinganines can be reduced to sphinganines which can be further converted 4-hydroxysphinganines (phytosphingosines). These LCB molecules can be N-acylated to generate ceramides. In mammals, the sphinganine moiety of ceramides can be converted to a sphingosine moiety (having a *cis* 4–5 double bond) and the corresponding ceramides species can be hydrolyzed by ceramidases to yield free sphingosines. Ceramides can also serve as precursors for synthesis of sphingomyelins and other complex sphingolipids such as gangliosides in mammals, and inositol-containing sphingolipids in yeast and plants [3]. Notably, LCB molecules can also be sequentially methylated to produce N-methyl-, N,N-dimethyl- and N,N,N-trimethyl-LCBs (TMLCB) in mammalian cells [4]. Moreover, under certain conditions the serine palmitoyltransferase can use alanine and glycine to produce neurotoxic LCBs without the archetypal C(1) hydroxyl group [5]. Taken together, LCB species can have different hydrocarbon chain lengths, and be classified into numerous subgroups having distinct structural attributes that span different numbers and positions of oxygen atoms and double bonds [6]. To understand how LCB metabolism and level of individual LCB species impinge upon physiological and pathophysiological processes warrants sensitive and specific assays for monitoring these molecules.

Mass spectrometry (MS)-based lipid analysis affords sensitive characterization of lipid molecules [7–9]. Strategies for analyzing lipids can be classified as either shotgun lipidomics, utilizing direct infusion MS, or as liquid chromatography (LC)-MS-based lipidomics. LCBs are typically analyzed using LC-MS/MS [10,11]. Such routines support detection and quantification of LCB species based on specific retention times and multiple reaction monitoring (MRM) of pre-defined precursor/fragment ion pairs that serve to enhance the specificity and sensitivity of the analysis. Notably, fragmentation of LCB species typically yield several fragment ions [10,11] that in principle should be monitored for accurate quantification and identification with high fidelity. LC-MS/MS-based workflows have primarily been implemented using low resolution triple quadrupole or ion trap-quadrupole instruments due to their high analytical sensitivity and acquisition speed. Such LC-MS/MS-based routines can be highly sensitive but their analytical specificity can potentially be compromised by substantial levels of interfering chemical noise that arise because of the low resolution ion detection inherent to the instrumentation. Moreover, LC-MS/MS-based approaches typically use relatively high flow rates (300–1000 μl/min) and electrospray ionization with high voltage and temperature settings (5 kV, 400°C) that promote in-source fragmentation of labile molecules such LCB species.

High resolution MS provides an alternative approach for monitoring LCB species. Mass spectrometers such as hybrid quadrupole time-of-flight (QqTOF) and Orbitrap-based machines provide high analytical specificity due to their high resolution ion detection capabilities [12,13]. In MS/MS mode these instruments support simultaneous detection of all fragment ions without the penalty of increased acquisition time as on triple quadrupole and ion trap instruments. This analytical attribute can be harnessed for designing sensitive and specific parallel reaction monitoring (PRM) assays where all fragment ions from a precursor ion are monitored simultaneously and at high analytical specificity supporting high fidelity identification [14,15]. Such a strategy could in principle be used for simultaneous monitoring of the multiple fragment ions released from LCB molecules. Similar to LC-MS-based workflows, high resolution lipidomics platforms can also suffer from in-source fragmentation of labile LCB species (S1 and S2 Figs) which can bias quantification. These caveats can be overcome if in-source fragmentation of LCB analytes could be eliminated.

**Fig 1:**
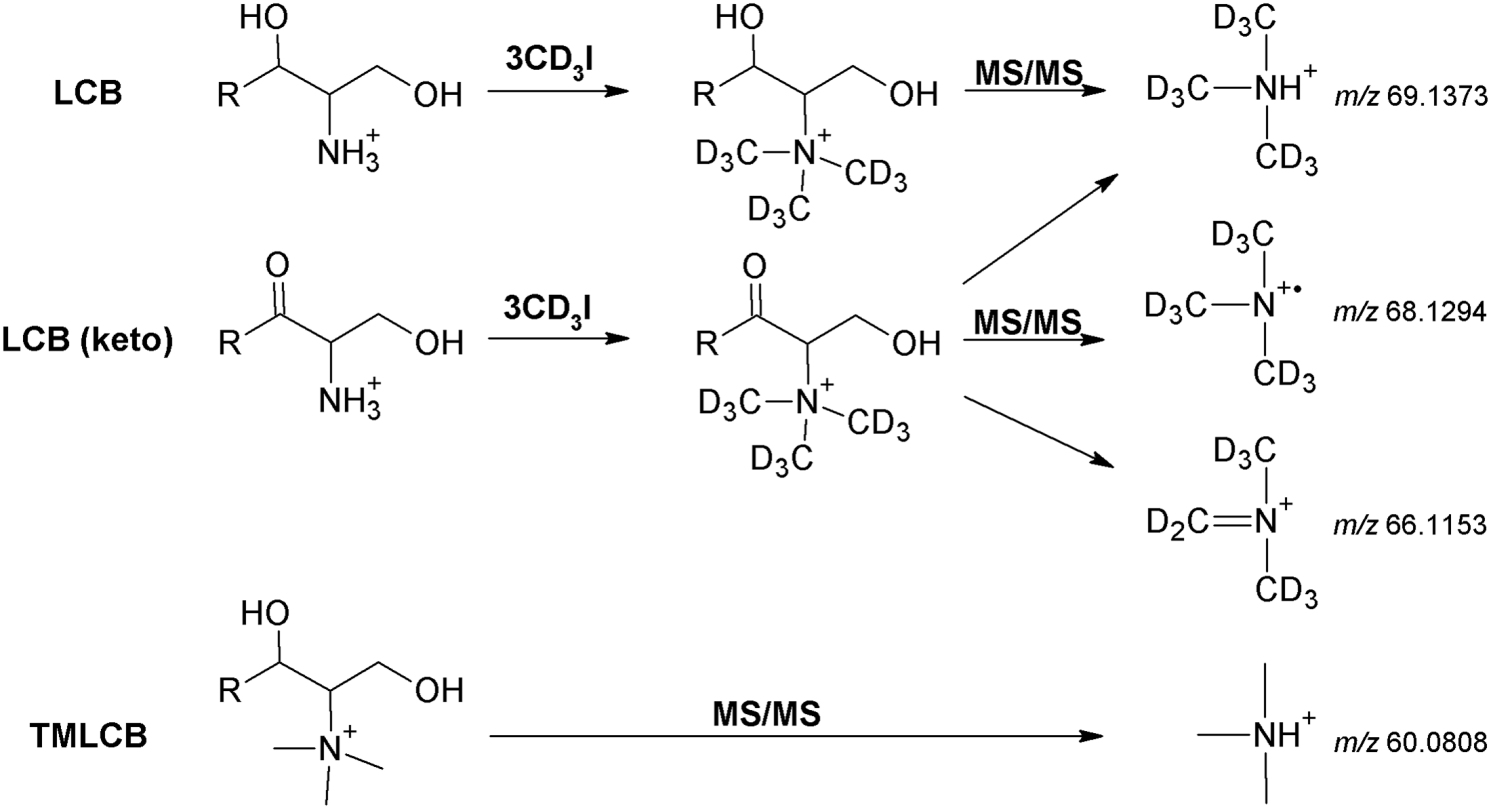
Outline of the mass-tag strategy used for monitoring of LCB species. LCB analytes are chemically derivatized using CD_3_I to produce TMLCB-like molecules having a precursor ion mass offset 9 Da. Upon fragmentation (MS/MS), derivatized LCBs ((CD_3_)_3_-LCB) produce TMLCB-like fragment ions also having a 9 Da mass offset. Note that fragmentation of derivatized 3-ketosphinganine (denoted LCB(keto)) also yields two structure-specific fragment ions.

Here we describe a high resolution shotgun lipidomics method that affords simple, sensitive and specific monitoring of LCB species. The method employs a specific “mass-tag” strategy [16] where LCB analytes are chemically derivatized using deuterated methyliodide (CD_3_I) to produce trimethylated derivatives having a positively charged quaternary amine group. These trimethylated LCB analytes do not undergo in-source fragmentation and yield a characteristic trimethylaminium fragment ion (Fig 1) that enables their sensitive and quantitative profiling using a complementary PRM assay executed using a QqTOF mass spectrometer equipped with a robotic nanoelectrospray ion source. Notably, this mass-tag strategy enabled, for the first time, specific monitoring of endogenous 3-ketosphinganine molecules and distinguishing them from more abundant isomeric sphingosine species based on the release of two structure-specific fragment ions. To document the efficacy of the methodology we report on an in-depth characterization of the LCB composition of yeast mutants with defective sphingolipid metabolism and the absolute levels of LCBs in mammalian cells. The methodology presented herein is generic, applicable to other lipidomics platforms and can serve as an additional routine in the expanding palette of mass spectrometric methods available for lipidome-wide quantification [17–19] and for functional studies of sphingolipid metabolism.

## Materials and Methods

### Annotation of lipid species

LCB species are annotated by sum composition: LCB <number of carbon atoms in the long-chain base >:<number of double bonds in the long-chain base>;<number of oxygen atoms in the long-chain base>. For example LCB 18:1;2 denotes a C_18_-sphingosine having one double bond and two oxygen atoms (equivalent to two hydroxyl groups). 3-ketophinganine molecules are denoted by the suffix (keto). For example, LCB 18:1;2(keto) denotes C_18_-3-ketophinganine having one double bond and two oxygen atoms (equivalent to one hydroxyl group and one ketone group featuring a double bond).

### Chemicals and lipid standards

Synthetic lipid standards were purchased from Avanti Polar Lipids and Larodan Fine Chemicals. CD_3_I was purchased from Cambridge Isotope Laboratories. All other chemicals and solvents were purchased from Sigma-Aldrich and Rathburn Chemicals.

### Yeast strains and standard culture conditions

The following strains were used: wild-type *S*. *cerevisiae* BY4742 (MATα *his3*Δ*1 leu2*Δ*0 lys2*Δ*0 ura3*Δ*0*), and congenic *sur2*Δ::*KanMX* and *elo3*Δ::*KanMX* deletion strains obtained from EUROSCARF. These strains were grown to mid-exponential phase (OD_600_ 1.0) at 20, 30 and 35°C in a synthetic defined medium containing 2% glucose as carbon source. Yeast cells were harvested by centrifugation, frozen in liquid nitrogen and stored at -80°C.

### Lipid extraction of yeast at 4°C

Yeast cell pellets were resuspended in 155 mM ammonium acetate and disrupted using glass beads. Yeast cell lysates corresponding to 0.4 OD_600_ units per 200 μl were spiked with 35 pmol C_17_-sphingosine (denoted LCB 17:1;2). The samples were subsequently extracted with 990 μl chloroform/methanol (15:1, V/V) for 90 min as previously described [17,20]. The lower organic phase was collected and subjected to vacuum evaporation. Lipid extracts were dissolved in 100 μl chloroform/methanol (1:2, V/V).

### Mammalian cell cultivation

HeLa cells were grown in RPMI media (Life Technologies) with 6% fetal calf serum and antibiotics at 37°C in a humidified atmosphere with 5% CO_2_. HeLa cells were trypsinized, centrifuged and resuspended in 155 mM ammonium acetate. One million cells were transferred to a new tube, spun down and washed twice in 155 mM ammonium acetate. The cell pellet was resuspended in 500 μl 155 mM ammonium acetate, snap-frozen in liquid nitrogen and stored at -80°C.

### Lipid extraction of HeLa cells at 4°C

Sample aliquots corresponding to 5∙10^5^ HeLa cells per 200 μl were spiked with 90 pmol C_17_-sphingosine (denoted LCB 17:1;2). The samples were subsequently extracted with 990 μl chloroform/methanol (10:1, V/V) for 90 min as previously described [18]. The lower organic phase was collected and evaporated. The lipid extract was dissolved in 100 μl chloroform/methanol (1:2, V/V).

### Chemical methylation

Synthetic lipid standards and lipid extracts (50 μl) were loaded in a 1.1 ml glass vials (La-Pha-Pack GmbH) and subjected to vacuum evaporation. Samples were dissolved in 100 μl CD_3_I (or CH_3_I) and placed in a thermomixer for 2.5 h (90°C, 600 rpm). The reaction was terminated by evaporation.

### Mass spectrometric analysis using a QqTOF instrument

Derivatized samples were dissolved in 50 μl chloroform/methanol/isopropanol (1:2:4, V/V/V) containing 7.5 mM ammonium acetate. Samples (18 μl) were loaded in a 96-well plate (Eppendorf AG) and covered with aluminum foil. Samples were analyzed in positive ion mode on a QSTAR Pulsar-*i* instrument (AB Sciex) equipped with a TriVersa NanoMate (Advion Biosciences), as previously described [21]. CD_3_I-derivatized LCB species were monitored by PRM using ten TOF MS/MS acquisitions (S1 Table). For all acquisitions a TOF MS/MS spectrum was recorded from *m/z* 50 to 80, the analytical quadrupole Q_1_ was operated at unit mass resolution, accumulation time was 1 s and fragment ion enhancement at *m/z* 69 [22].

### Data processing

**Fig 2:**
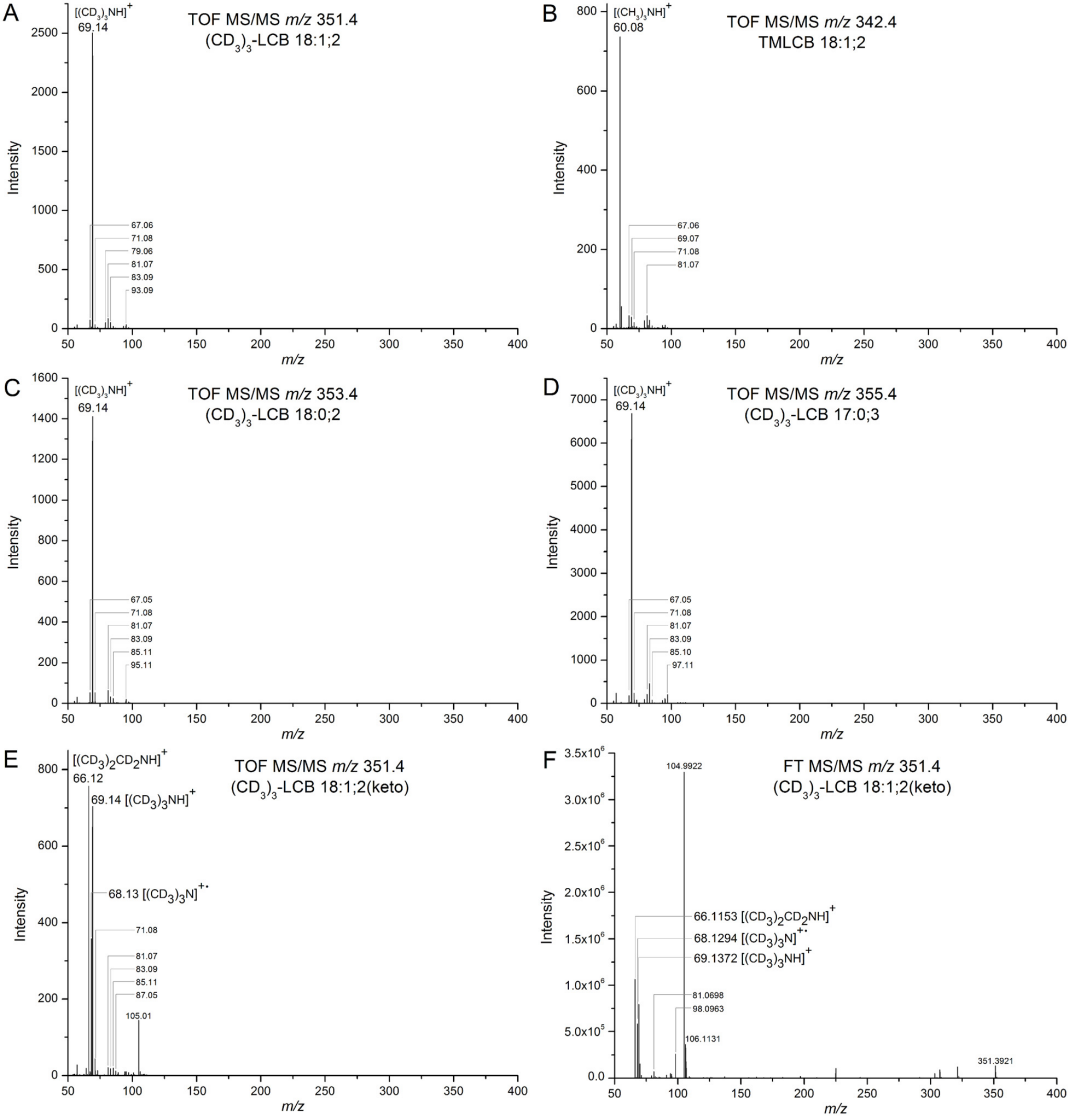
Structural characterization of CD_3_I-derivatized LCB species and trimethyl-C_18_-sphingosine. **(A)** TOF MS/MS spectrum of derivatized C_18_-sphingosine ((CD_3_)_3_-LCB 18:1;2) using collision energy at 30 eV. **(B)** TOF MS/MS spectrum of trimethyl-C_18_-sphingosine (TMLCB 18:1;2) using collision energy at 30 eV. **(C)** TOF MS/MS spectrum of derivatized C_18_-sphinganine ((CD_3_)_3_-LCB 18:0;2) using collision energy at 35 eV. **(D)** TOF MS/MS spectrum of derivatized C_17_-4-hydroxysphinganine ((CD_3_)_3_-LCB 17:0;3) using collision energy at 35 eV. **(E)** TOF MS/MS spectrum of derivatized C_18_-3-ketosphinganine ((CD_3_)_3_-LCB 18:1;2(keto)) using collision energy at 30 eV. **(F)** FT MS/MS spectrum of derivatized C_18_-3-ketosphinganine ((CD_3_)_3_-LCB 18:1;2(keto)) using a relative collision energy at 55%. The mass resolution of the fragment ion *m/z* 66.1153 is 434,420 (full width at half maximum). All TOF MS/MS spectra were acquired using ion enhancement at *m/z* 69.14.

**Fig 3:**
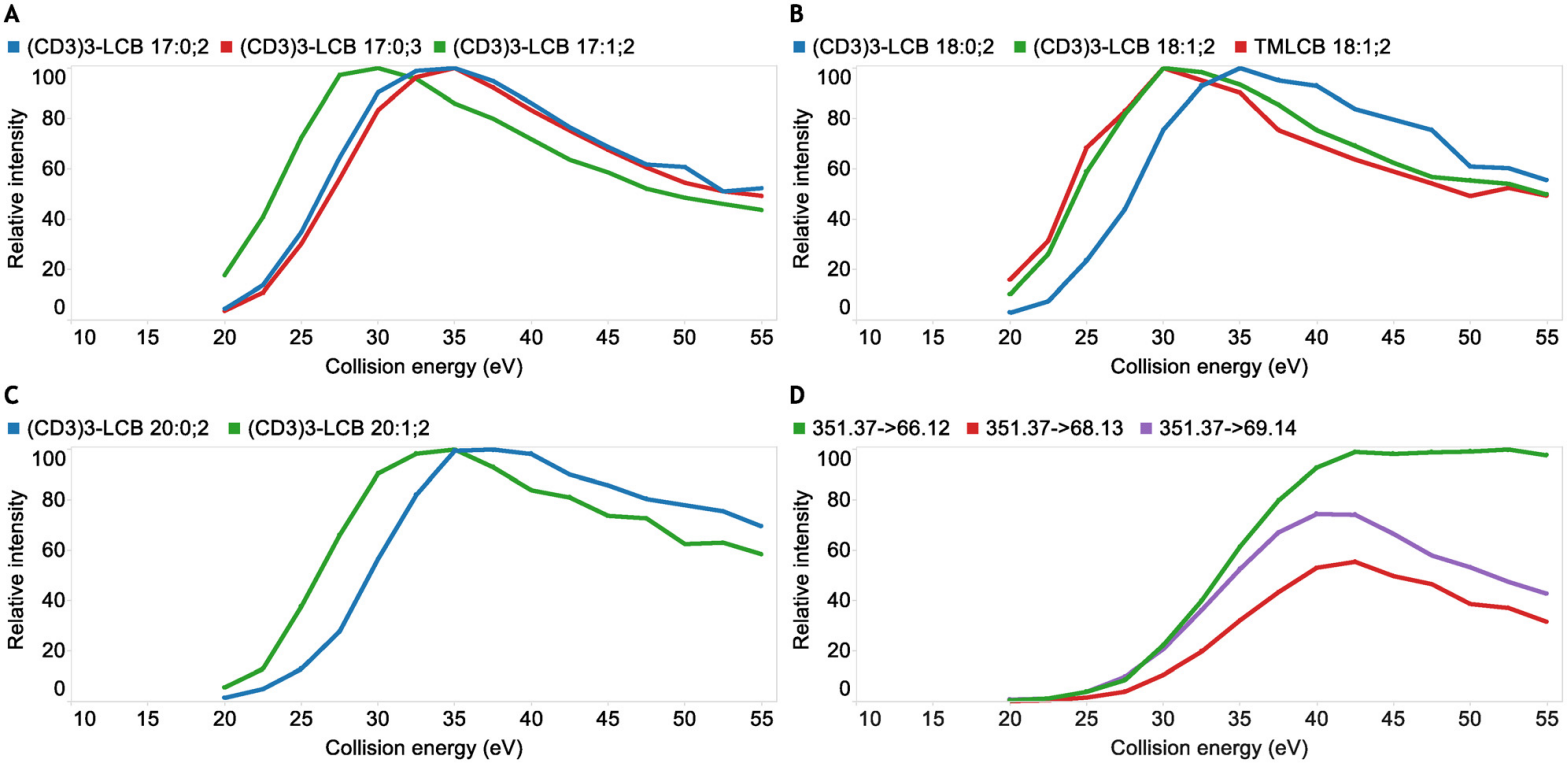
Optimization of collision energy settings for PRM of derivatized LCB species. **(A)** Relative intensity of fragment ion *m/z* 69.14 as function of collision energy for C_17_-sphingosine (denoted (CD_3_)_3_-LCB 17:1;2), C_17_-sphinganine ((CD_3_)_3_-LCB 17:0;2) and C_17_-4-hydroxysphinganine ((CD_3_)_3_-LCB 17:0;3). **(B)** Relative intensity of fragment ion *m/z* 69.14 as function of collision energy for C_18_-sphingosine ((CD_3_)_3_-LCB 18:1;2) and C_18_-sphinganine ((CD_3_)_3_-LCB 18:0;2), and of fragment ion *m/z* 60.08 trimethyl-C_18_-sphingosine (TMLCB 18:1;2). **(C)** Relative intensity of fragment ion *m/z* 69.14 as function of collision energy for C_20_-sphingosine ((CD_3_)_3_-LCB 20:1;2) and C_20_-sphinganine ((CD_3_)_3_-LCB 18:0;2). **(D)** Relative intensities of fragment ions *m/z* 66.12, *m/z* 68.13 and *m/z* 69.14 as function of collision energy for CD_3_I-derivatized C_18_-3-ketosphinganine.

Mass spectral data were extracted from proprietary data files using LipidView software [21] and further processed using SAS Enterprise Guide and Tableau Desktop for quantification of LCBs and data visualization, respectively [23]. An intensity threshold of 15 counts was implemented based on manually assessing the minimum intensity required to produce a fragment ion peak. A dedicated algorithm was implemented to estimate the proportion of *m/z* 69.14 fragment ion intensity derived from isomeric (CD_3_)_3_-LCB X:1;2(keto) and (CD_3_)_3_-LCB X:1;2 analytes (where X = {16,18,20}). Both (CD_3_)_3_-LCB X:1;2(keto) and (CD_3_)_3_-LCB X:1;2 analytes release the fragment ion *m/z* 69.14. (CD_3_)_3_-LCB X:1;2(keto) analytes also release fragment ions *m/z* 66.12 and *m/z* 68.13 (Fig 2E). For derivatized C_18_-3-ketosphinganine ((CD_3_)_3_-LCB X:1;2(keto)) the fragment ion intensity ratio of *m/z* 69.14 and *m/z* 66.12 was experimentally determined to be 0.955, at a collision energy of 30 eV as used for the PRM assay (Fig 3D). This fragment ion intensity ratio was used to interrelate the intensities of the two (CD_3_)_3_-LCB X:1;2(keto)-derived fragment ions using Eq (1):

$${\text{I}}_{\text{(CD3)3}-\text{LCB X:1;2(keto),}m/z\text{ 64.14}}=\text{0.955}\times {\text{I}}_{\text{(CD3)3}-\text{LCB X:1;2(keto),}m/z\text{ 66.12 }}$$ (1)

where I_(CD3)3-LCB X:1;2(keto),m/z 69.14_ and I_(CD3)3-LCB X:1;2(keto),m/z 66.12_ are the intensities of the fragment ions *m/z* 69.14 and 66.12 derived from a (CD_3_)_3_-LCB X:1;2(keto) species. Consequently, in complex sample matrices the proportion of *m/z* 69.14 fragment ion intensity derived from (CD_3_)_3_-LCB X:1;2 analytes can be estimated using Eq (2):

$${\text{I}}_{\text{(CD3)3-LCB X:1;2,}m/z\text{ 69.14}}{\text{ = I}}_{\text{TOTAL,}m/z\text{ 69.14}}\text{ - 0.955}\times {\text{I}}_{\text{(CD3)3-LCB X:1;2(keto),}m/z\text{ 66.12}}$$ (2)

where I_TOTAL,m/z 69.14_ is the recorded intensity of the fragment ion *m/z* 69.14, I_(CD3)3-LCB X:1;2(keto),m/z 66.12_ is the intensity of the (CD_3_)_3_-LCB X:1;2(keto)-specific fragment ion at *m/z* 66.12 and I_(CD3)3-LCB X:1;2,m/z 69.14_ is the estimated intensity of the (CD_3_)_3_-LCB X:1;2 fragment ion at *m/z* 69.14. In addition, the following Eq (3) can be used to estimate the fragment ion intensity derived from (CD_3_)_3_-LCB X:1;2(keto) analytes in complex sample matrices:

$${\text{I}}_{\text{(CD3)3-LCB X:1;2(keto)}}{\text{ = I}}_{\text{(CD3)3-LCB X:1;2(keto),}m/z\text{ 68.13}}\text{ + (1+0.955)}\times {\text{I}}_{\text{(CD3)3-LCB X:1;2(keto),}m/z\text{ 66.12}}$$ (3)

where I_(CD3)3-LCB X:1;2(keto)_ is the estimated sum of all monitored (CD_3_)_3_-LCB X:1;2(keto) fragment ions. The above-listed equations were implemented in an algorithm to estimate the fragment ion intensities derived from (CD_3_)_3_-LCB X:1;2 and (CD_3_)_3_-LCB X:1;2(keto) analytes. Using this algorithm a dataset with molar abundances of LCB species was obtained by normalizing the fragment ion intensities of derivatized LCB species relative to the fragment intensity of the internal standard C_17_-sphingosine and multiplying with the spike amount of the internal standard [21]. The molar abundances of LCB species were subsequently normalized to the extracted sample amount and to the total levels of all LCB species to determine the stoichiometry of all monitored LCB species (expressed as mol%). Molar abundances of LCB species detected in yeast and HeLa cells are available in S2 and S3 Tables, respectively.

### Mass spectrometric analysis using an Orbitrap Fusion instrument

CD_3_I-derivatized C_18_-3-ketosphinganine was dissolved in chloroform/methanol/isopropanol (1:2:4, V/V/V) containing 7.5 mM ammonium acetate, loaded in a 96-well plate and analyzed in positive ion mode on an Orbitrap Fusion Tribrid (Thermo Scientific) equipped with a TriVersa NanoMate (Advion Biosciences). Fourier transform (FT) MS/MS spectra were recorded using maximum injection time of 100 ms, automated gain control at 5e4, four microscans and a target resolution of 240,000 [13].

## Results and Discussion

### A mass-tag strategy for monitoring LCB species as TMLCB-like molecules

We recently developed a shotgun lipidomics method for sensitive and quantitative analysis of aminoglycerophospholipids based on a specific mass-tag strategy that uses deuterated methyliodide (CD_3_I) to trimethylate amine groups [16]. To evaluate if this mass-tag strategy is also applicable for profiling LCB species, which also contain an amine group (Fig 1), we tested whether LCBs could be converted to trimethylated derivatives (denoted (CD_3_)_3_-LCB)). TOF MS analysis of derivatized synthetic LCBs demonstrated that trimethylate LCB analytes were easily produced and detected as precursor ions having a systematic and specific mass offset of 9.06 Da as compared to equivalent TMLCBs. Inspection of TOF MS data showed no signs of in-source fragmentation ascribed to neutral loss of H_2_O, as observed for underivatized LCB species (S1 Fig). In addition, no detection of a characteristic trimethylaminium fragment ion was observed (see discussion below). Furthermore, no in-source fragmentation was observed when analyzing CD_3_I-derivatized LCB species using an Orbitrap Fusion mass spectrometer (data not shown). We also evaluated whether the CD_3_I derivatization would improve the ionization efficiency of LCB analytes. To this end, we infused equimolar mixtures of underivatized and derivatized C_18_-sphingosine. This evaluation showed that the intensity of the CD_3_I-derivatized C_18_-sphingosine was 3.5-fold more abundant as compared to the underivatized LCB analyte (S2 Fig). Based on these results we conclude that CD_3_I-derivatization of LCBs to TMLCB-like analytes stabilizes LCB analytes and prevents their in-source fragmentation, and also improves their ionization efficiency. We note that we choose to use CD_3_I instead of CH_3_I for derivatization as mammalian cells, and potentially also yeast cells, can produce endogenous TMLCB species [4] that could potentially compromise the analytical specificity of the methodology.

Structural characterization of synthetic and CD_3_I-derivatized LCBs by TOF MS/MS demonstrated that all analytes release an abundant deuterated trimethylaminium ([(CD_3_)_3_NH]^+^) fragment ion with *m/z* 69.14 (Fig 2 and S3 Fig). Similar to precursor ions, this fragment ion is also offset by 9.06 Da as compared to the TMLCB-derived trimethylaminium fragment ion with *m/z* 60.08 ([(CH_3_)_3_NH]^+^) (Fig 2B). In addition, all derivatized LCB analytes also released clusters of low abundant fragment ions having odd nominal masses with intra-cluster mass differences of 2.02 Da and inter-cluster mass differences of 12.00 Da. These clusters of low abundant ions correspond to fragments having different numbers of double bonds and carbon atoms. Comparing the pattern of these low abundant fragment ions did not reveal any fragments that could provide structure-specific information on individual LCB molecules. However, fragmentation of CD_3_I-derivatized C_18_-3-ketosphinganine showed the expected deuterated trimethylaminium fragment ion with *m/z* 69.14 ([(CD_3_)_3_NH]^+^) as well as two abundant fragment ions at *m/z* 66.12 and *m/z* 68.13, which could not be observed for other derivatized LCB species. These fragment ions at *m/z* 66.12 and *m/z* 68.13 corresponds to deuterated N,N-dimethyl-N-methyleneanaminium ([(CD_3_)_2_(CD_2_)N]^+^) and a deuterated trimethylanaminium radical ([(CD_3_)_3_N]^+•^), respectively. To confirm the identities of these structure-specific fragment ions we derivatized C_18_-3-ketosphinganine using CH_3_I and subjected the trimethylated derivative to TOF MS/MS analysis (S4 Fig). This analysis demonstrated detection of fragment ions at *m/z* 58.07 and *m/z* 59.07 corresponding to N,N-dimethyl-N-methyleneanaminium ([(CH_3_)_2_(CH_2_)N]^+^) and a trimethylanaminium radical ([(CH_3_)_3_N]^+•^), respectively. To further support the identities of the 3-ketosphinganine-specific fragment ions we analyzed the CD_3_I-derivatized C_18_-3-ketosphinganine using high resolution FT MS/MS analysis on an Orbitrap Fusion mass spectrometer (Fig 2F). This analysis confirmed the fragment ion identities as deuterated N,N-dimethyl-N-methyleneanaminium ([(CD_3_)_2_(CD_2_)N]^+^) and trimethylanaminium radical ([(CD_3_)_3_N]^+•^), both with a mass accuracy of 0.3 ppm. Based on the abovementioned results we conclude that CD_3_I-derivatized LCB species can be identified based on their release of an abundant deuterated trimethylaminium fragment ion, and that 3-ketosphinganines can be distinguished from isomeric sphingosines or monounsaturated sphinganines based on the release of the two structure-specific fragments ions with *m/z* 66.12 and 68.13.

### Design and optimization of a PRM assay for quantitative LCB profiling

To quantitatively monitor LCB species in biological matrices we designed a PRM assay. This PRM assay comprises ten multiplexed TOF MS/MS acquisitions corresponding to CD_3_I-derivatives of commonly occurring LCB species in yeast and mammalian cells having a total of 16, 18 or 20 carbon atoms, 2 or 3 oxygen atoms and 0 or 1 double bond (S1 Table). In addition, the PRM assay also featured a transition for the internal standard C_17_-sphingosine. For each acquisition a TOF MS/MS spectrum was recorded in the range of *m/z* 50 to 80 in order to support parallel detection of multiple fragment ions released from the same precursor ion (i.e. 3-ketosphinganine analytes).

For optimal detection of LCB precursor/fragment ion pairs we systematically evaluated the collision energy dependency for release of the trimethylaminium fragment ion (Fig 3). This evaluation was performed for eight representative LCB species having different chemical structures. Analysis of derivatized sphingosines demonstrated that the optimal collision energy for detection of *m/z* 69.14 was around 30–32.5 eV. For comparison we also examined underivatized trimethyl-C_18_-sphingosine and found that the optimal collision energy for detection of *m/z* 60.08 was 30 eV (Fig 2B). This result shows that the deuterium atoms in the CD_3_I-derivatized sphingosine analytes do not alter the fragmentation properties of CD_3_I-derivatized sphingosine species. The evaluation of optimal collision energy for detection of *m/z* 69.14 released from derivatized sphinganine and 4-hydroxysphinganine species showed optimal collision energies around 35 eV, approximately 5 eV higher as compare to their sphingosine counterparts. This result shows that optimal detection of the deuterated trimethylaminium fragment ion is dependent on the structural attributes of the derivatized LCB molecule and that optimal collision energies should be meticulously evaluated in order to optimize PRM performance.

Assessing the impact of collision energy on the intensities of fragment ions released from derivatized C_18_-3-ketosphinganine showed, surprisingly, that optimal detection of *m/z* 66.12, *m/z* 68.13 and *m/z* 69.14 was achieved at collision energies of 42.5, 42.5 and 40 eV, respectively. This is approximately 10 eV higher as compared to the isomeric C_18_-sphingosine analyte. Notably, at a collision energy of 30 eV the intensity ratio of the two most abundant 3-ketosphinganine-derived fragment ions at *m/z* 69.14 and *m/z* 66.12 was 0.955 (Fig 3D). This intensity ratio was constant across multiple evaluations (data not shown) and was used for estimating the relative proportion of isomeric 3-ketosphinganine, sphingosine and monounsaturated sphinganine species in biological matrices. For example, no detection of *m/z* 66.12 (and also no detection of *m/z* 68.13) shows that 100% of the intensity of *m/z* 69.14 derive from a (CD_3_)_3_-LCB 18:1;2 analyte. In contrast, an intensity ratio of 0.955 between *m/z* 69.14 and 66.12 shows that 100% of the *m/z* 69.14 intensity derive from a (CD_3_)_3_-LCB 18:1;2(keto) analyte. For intermediate intensity ratios the proportion of isomeric (CD_3_)_3_-LCB 18:1;2 and (CD_3_)_3_-LCB 18:1;2(keto) analytes can be estimated using an algorithm that employs the intensity ratio (see Materials and Methods for details). We note here that the designed PRM assay with ten TOF MS/MS acquisitions effectively equals nineteen precursor/fragment ion transitions given that three additional fragment ions are monitored for potential C_16_-, C_18_- and C_20_-3-ketosphinganine analytes (S1 Table). The intensities for all transitions were extracted from proprietary mass spectral data files using LipidView software [21] and further processed using SAS Enterprise Guide and Tableau Desktop for quantification and visualization, respectively [23].

### Evaluation of dynamic quantification range of PRM assay

Having designed and optimized the PRM assay we next evaluated its dynamic quantification range for profiling of LCB species in yeast and mammalian cells. As a premise for this evaluation we first examined the CD_3_-labeling efficiency. To this end, we spiked cell lysates of *S*. *cerevisiae* with equimolar amounts of synthetic C_17_-sphingosine (denoted LCB 17:1;2) and trimethyl(CH_3_)_3_-sphingosine (denoted TMLCB 18:1;2), performed lipid extraction, derivatized the lipid extracts with CD_3_I, and analyzed samples using PRM. Comparing fragment ion intensities of derivatized (CD_3_)_3_-LCB 17:1;2 and TMLCB 18:1;2 showed that 98% of C_17_-sphingosine was labeled (S5 Fig). This high labeling efficiency corroborates our previous results obtained with phosphatidylethanolamine [16].

**Fig 4:**
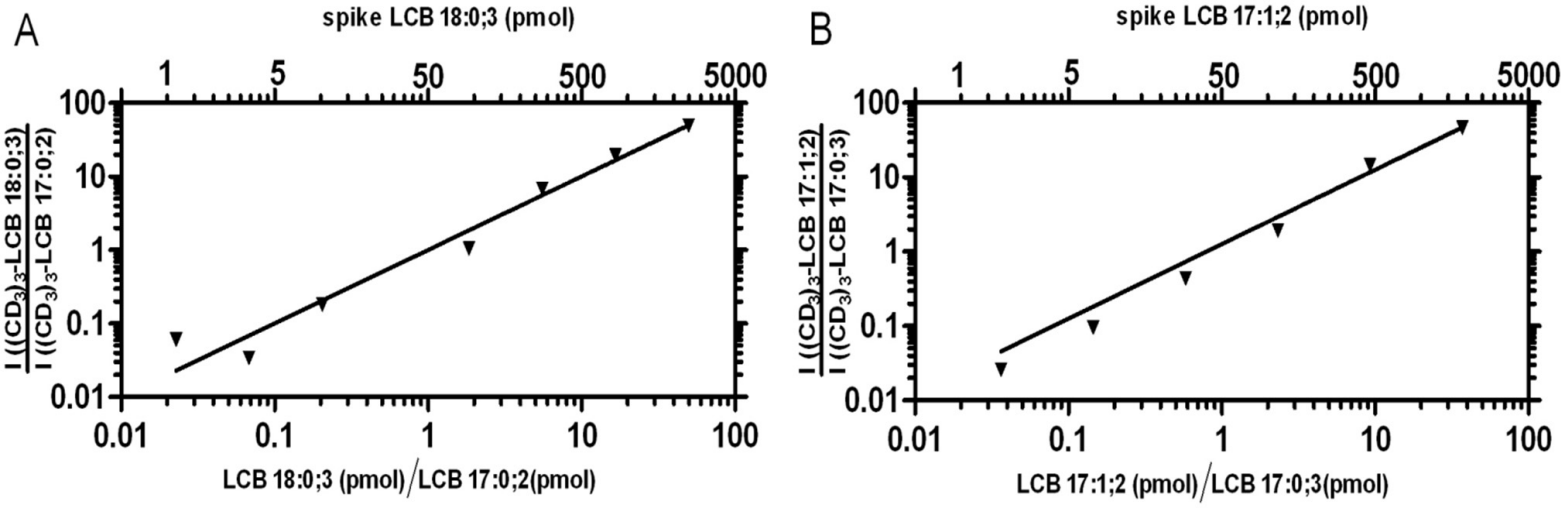
Dynamic quantification range of the PRM assay for quantitative LCB profiling. **(A)** Synthetic C_18_-4-hydroxysphinganine (denoted LCB 18:0;3) was titrated relative to a constant amount of synthetic C_17_-sphinganine (LCB 17:0;2), and spiked into *S*. *cerevisiae sur2*Δ cell lysates followed by lipid extraction. Lipid extracts were derivatized using CD_3_I and analyzed by PRM. The upper x-axis shows the absolute spike amount of LCB 18:0;3 (1–2457 pmol). The lower x-axis shows the spike amount of LCB 18:0;3 relative to LCB 17:0;2. The y-axis shows the intensity ratio of the transition *m/z* 369.4→*m/z* 69.14 (monitoring (CD_3_)_3_-LCB 18:0;3) and *m/z* 339.4→*m/z* 69.14 (monitoring (CD_3_)_3_-LCB 17:0;2). Depicted values derive from two replicate analyses. The line indicates the linear function with slope 1. **(B)** Synthetic C_17_-sphingosine (denoted LCB 17:1;2) was titrated relative to a constant amount synthetic C_17_-4-hydroxysphinganine (LCB 17:0;3), and spiked into HeLa cell lysates followed by lipid extraction. Lipid extracts were derivatized using CD_3_I and analyzed by PRM. The upper x-axis shows the absolute spike amount of LCB 17:1;2 (2–1833 pmol). The lower x-axis shows the spike amount of LCB 17:1;2 relative to LCB 17:0;3. The y-axis shows the intensity ratio of the transition *m/z* 337.4→*m/z* 69.14 (monitoring (CD_3_)_3_-LCB 17:1;2) and *m/z* 355.4→*m/z* 69.14 (monitoring (CD_3_)_3_-LCB 17:0;3). Depicted values are the average of two replicate analyses. The line indicates the linear function with slope 1.

Having established that the CD_3_I derivatization is efficient, we next evaluated the dynamic quantification range of the PRM assay for yeast samples. To this end, we analyzed a dilution series of two synthetic LCB standards where one species, C_18_-4-hydroxysphinganine (denoted LCB 18:0;3), was titrated relative to a constant amount of C_17_-sphinganine (LCB 17:0;2). This dilution series was spiked into a cell lysates of the *S*. *cerevisiae* strain *sur2*Δ which cannot synthesize 4-hydroxysphinganines and, hence, allows specific detection of LCB 18:0;3 in a biological sample matrix. Yeast cell lysates spiked with mixtures of the synthetic LCB standards were subjected to lipid extraction followed by CD_3_I derivatization and PRM (each lipid extract was injected twice). To evaluate the dynamic quantification range of the PRM assay, we plotted the intensity ratio between the two CD_3_I-derivatized LCB species as a function of both their molar ratio and the spike amount of C_18_-4-hydroxysphinganine (Fig 4A). This assessment demonstrated that the response of the PRM assay was linear with a slope value of approximately one across three orders of magnitude. The limit of quantification of the PRM assay for profiling C_18_-4-hydroxysphinganine (LCB 18:0;3) was approximately 1 pmol (corresponding to a molar ratio value of 0.02 in Fig 4A).

Similar results were obtained when validating the PRM assay for profiling LCB species in mammalian cells. For this evaluation we analyzed a dilution series of synthetic C_17_-sphingosine (denoted LCB 17:1;2) titrated relative to synthetic C_17_-4-hydroxysphinganine (LCB 17:0;3) spiked into a sample matrix of HeLa cells. This analysis again demonstrated that the response of the PRM assay was linear with a slope value of approximately one across three orders of magnitude for the analyte C_17_-sphingosine (LCB 17:1;2). The limit of quantification for monitoring LCB 17:1;2 was approximately 2 pmol (corresponding to a molar ratio value of 0.04 in Fig 4B). Based on this validation we conclude that the mass-tag strategy and the complementary QqTOF-based PRM assay affords sensitive and accurate analysis of LCB species in sample matrices of both yeast and mammalian cells.

### Quantitative profiling of LCB species in *S*. *cerevisiae*

**Fig 5:**
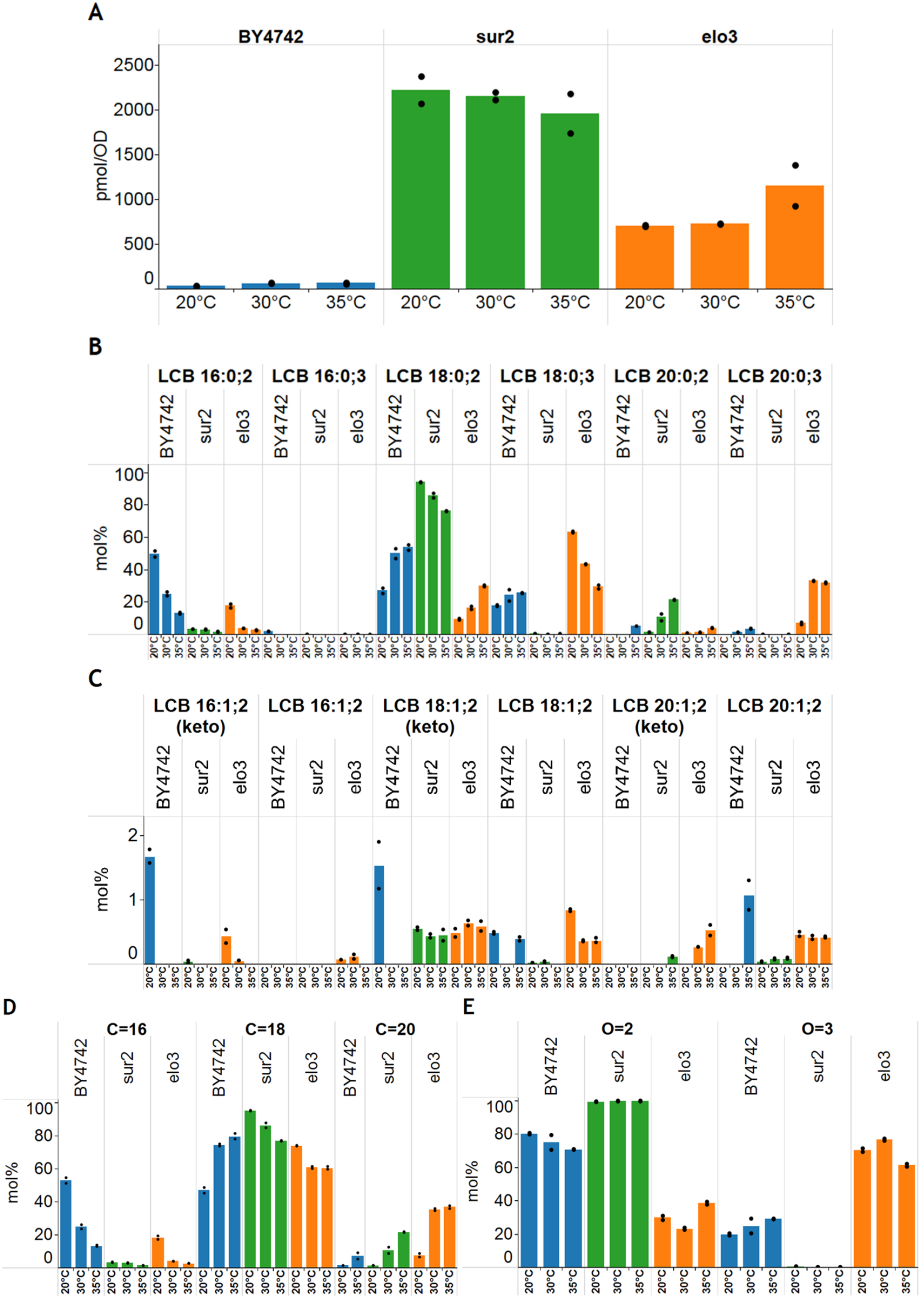
Profiling LCB species composition of *S*. *cerevisiae*. Wild-type BY4742, *sur2*Δ and *elo3*Δ were cultured at 20°C, 30°C and 35°C. Lipid extracts were prepared, derivatized using CD_3_I and analyzed by PRM. **(A)** Absolute levels of LCB species. **(B)** Molar abundances of sphinganine and 4-hydroxysphinganine species. **(C)** Molar abundances of 3-ketosphinganine and monounsaturated sphinganine species. **(D)** Combined molar abundances of C_16_-, C_18_- and C_20_-LCB species. **(E)** Combined molar abundances of all LCB species with two or three oxygen atoms. Data are expressed as average of two biological replicates analyzed by two repeated injections. The averages of the repeated injections are shown by black dots. Data is available in S2 Table.

As a test bed to demonstrate the efficacy of the mass-tag strategy for biochemical studies of sphingolipid metabolism we next investigated how different growth temperatures affects the LCB species composition of *S*. *cerevisiae* strains with and without defective *de novo* sphingolipid metabolism. To this end, we assayed the wild-type strain BY4742 and the deletion mutants *sur2*Δ, which is devoid of the C_4_-hydroxylase that catalyses the conversion of sphinganines to 4-hydroxysphinganines, and *elo3*Δ, which lacks an elongase activity involved in the production of C_26_ fatty acids utilized for ceramide synthesis. The three yeast strains were cultured at 20°C, 30°C and 35°C (two biological replicates per strain). Cell lysates of the yeast strains were spiked with the internal standards C_17_-sphingosine for quantification of endogenous LCB species. These cell lysates were subjected to lipid extraction followed by CD_3_I-derivatization and PRM (each lipid extract was injected twice). Collectively, this analysis revealed several important observations regarding both the performance of methodology and the biochemical impact of temperature and ablating the genes *SUR2* and *ELO3*. First, we observed that the *sur2*Δ deletion strain had the highest levels of LCB species across all growth temperatures (Fig 5A). At the growth temperatures of 20°C, 30°C and 35°C the total LCB levels were 2221, 2152 and 1959 pmol/OD, respectively. In comparison, the total levels of LCBs in *elo3*Δ cultured at 20°C, 30°C and 35°C were 3.2-, 3.0- and 1.7-fold less abundant as compared to the LCB levels in the *sur2*Δ strain (Fig 5A). The absolute level of the species C_18_-4-hydroxysphinganine in *elo3*Δ cultured at 30°C was 316 pmol/OD, which corresponds to levels obtained for this strain in previous analyses [17]. As expected, the wild-type strain BY4742 had the lowest levels of LCB species across all growth temperatures. At the growth temperature of 20°C, 30°C and 35°C the total LCB levels were 30, 63 and 65 pmol/OD, respectively. Taken together these results suggest that the high LCB levels in *sur2*Δ result from a lack of the C_4_-hydroxylase activity which impedes the synthesis of ceramide and down-stream sphingolipid species. The lower LCB levels in *elo3*Δ indicate a less perturbed rate of ceramide synthesis probably attributed the appropriate production of 4-hydroxysphinganines in this strain.

The analytical specificity of the methodology is highlighted by the specific detection of LCB species with only two oxygen atoms in the *sur2*Δ strain (Fig 5B and 5D). In addition, the efficacy of the methodology is also demonstrated by the specific detection and quantification of low abundant LCB species in wild-type yeast. Evaluating the stoichiometry of LCB species demonstrated that wild-type BY4742 accumulate primarily C_16_- and C_18_-sphinganine species (denoted LCB 16:0;2 and LCB 18:0;2 in Fig 5B), and lower amounts of C_18_-4-hydroxysphinganine (LCB 18:0;3). At the growth temperature of 35°C, the LCB 16:0;2, LCB 18:0;2 and LCB 18:0;3 species comprised 13, 54 and 26 mol% of all LCBs in wild-type BY4742. Reducing the growth temperature of BY4742 from 35°C to 20°C resulted in a systematic, and expected, decrease in the hydrocarbon chain length of LCBs from primarily C_18_- and C_20_-species to more C_16_-species (Fig 5B and 5D). Interestingly, the accumulation of primarily sphinganines (LCB 16:0;2 and LCB 18:0;2) in wild-type BY4742 suggests that the C_4_-hydroxylase Sur2p can also be rate-limiting for *de novo* LCB synthesis in yeast. A temperature-dependency on LCB hydrocarbon chain length was also observed for *sur2*Δ and *elo3*Δ, although both strains overall synthesize higher levels of C_18_- and C_20_-LCB species as compared to wild-type BY4742 (Fig 5D). Changing the growth temperature had only minor effects on the total number of oxygen atoms in the LCB species of BY4742 and *elo3*Δ (Fig 5E).

The PRM assay also featured transitions for monitoring LCB species having one double bond. Such LCB species can comprise isomeric 3-ketosphinganines, sphingosines having a double bond located between carbon atom 4 and 5, and monounsaturated sphinganines having a double bond located elsewhere in the hydrocarbon chain. Such LCB molecules are typically not reported in the *S*. *cerevisiae* since this yeast cannot synthesize sphingosines and because 3-ketosphinganines are very low abundant and considered below the detection limit of most analytical methods. However, the *S*. *cerevisiae* serine palmitoyltransferase can in principle utilize monounsaturated acyl-CoAs in addition to its preferred substrate palmitoyl-CoA. Hence, *S*. *cerevisiae* should be able to synthesize monounsaturated sphinganines as well as isomeric 3-ketosphinganines. Evaluating the composition of monounsaturated LCB species in the three yeast strains showed the detection and quantification of primarily C_18_-3-ketosphinganine (denoted LCB 18:1;2(keto) in Fig 5C) and isomeric monounsaturated C_18_-sphinganine (LCB 18:1;2). Notably, *elo3*Δ also featured significant amounts of C_20_-3-ketosphinganine (LCB 20:1;2(keto)) and monounsaturated C_20_-sphinganine (LCB 20:1;2). This observation correlates with the higher levels of also C_20_-sphinganine and C_20_-4-hydroxysphinganine in this strain (Fig 5B). Taken together, these results demonstrate that the *S*. *cerevisiae* serine palmitoyltransferase can use palmitoleoyl (16:1)-CoA, stearoyl (18:0)-CoA and oleoyl (18:1)-CoA as substrates. Across all growth temperatures the total levels of 3-ketosphinganines and monounsaturated sphinganines were relatively constant for both *sur2*Δ and *elo3*Δ. In *sur2*Δ, 3-ketosphinganines and monounsaturated sphinganines represented on average 0.6 mol% and 0.08 mol%, respectively, of all monitored LCB molecules. In *elo3*Δ, 3-ketosphinganines and monounsaturated sphinganines constituted on average 1 mol% of all quantified LCBs. In comparison, for wild-type BY4742 we could only detect C_16_- and C_18_-3-ketosphinganines (LCB 16:1;2(keto) and LCB 18:1;2(keto)) when cultured at 20°C whereas monounsaturated C_18_- and C_20_-sphinganines (LCB 18:1;2 and LCB 20:1;2) could be detected at almost all growth temperatures (Fig 5C). In wild-type BY4742, 3-ketosphinganine and monounsaturated sphinganine species represented up to 3.4 mol% and 1.5 mol%, respectively, of all monitored LCB species. We note that no major changes where observed for the number of double bonds in LCB species when changing the growth temperature (data not shown). Based on the above-mentioned results we conclude that the mass-tag strategy and the complementary PRM assay is a powerful analytical routine supporting sensitive, specific and quantitative profiling of LCB species in yeast. Importantly, this methodology has enabled, for the first time, quantitative profiling of 3-ketosphinganines and monounsaturated sphinganines in the yeast *S*. *cerevisiae*.

### Quantitative profiling of LCB species in HeLa cells

**Fig 6:**
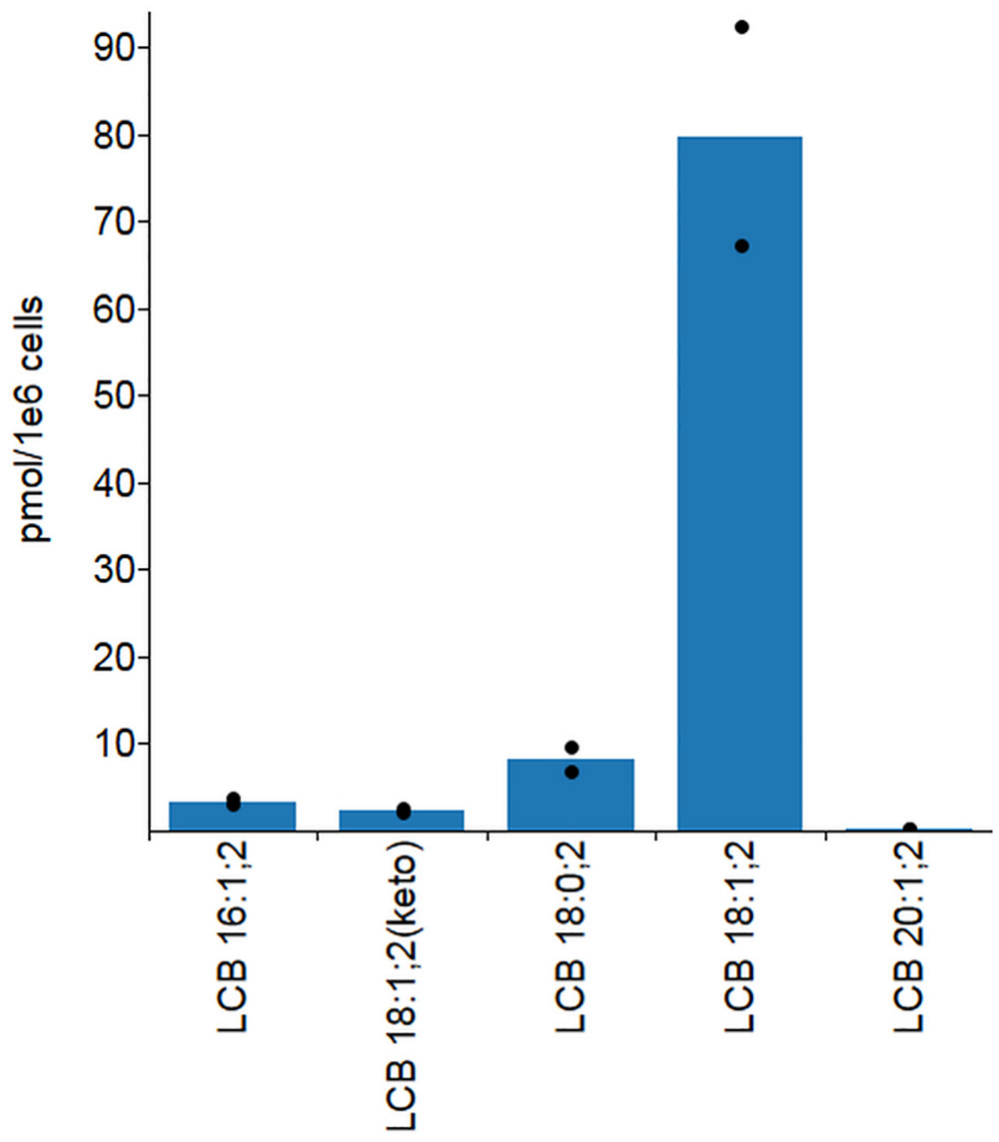
Quantitative profiling of LCB species in HeLa cells. Lysates of HeLa cells were spiked with internal standard C_17_-sphingosine, subjected to lipid extraction followed by CD_3_I-derivatization and PRM. Data are expressed as average of two replicate measurements indicated by black dots. Data is available in S3 Table.

Next we evaluated the efficacy of the methodology for profiling LCBs in mammalian cells. To this end, we spiked lysates of HeLa cells with the internal standard C_17_-sphingosine, subjected the cell lysates to lipid extraction followed by CD_3_I-derivatization and analysis by PRM (the lipid extract was analyzed twice). This analysis demonstrated that the most abundant LCB species in HeLa cells is LCB 18:1;2 and constitutes 80 pmol per 10^6^ cells (Fig 6). This LCB analyte most likely represents the common C_18_-sphingosine [1] but we note that the methodology cannot rule out the presence of a supposedly less common and isomeric monounsaturated C_18_-sphinganine. The abundance of 80 pmol C_18_-sphingosine per 10^6^ cells agrees well with previous analyses of fibroblasts and RAW264.7 cells having an estimated 24 to 105 pmol per 10^6^ cells [10,11]. Moreover, the analysis also showed the detection of less abundant LCB species corresponding to C_18_-sphinganine (LCB 18:0;2, 8 pmol/10^6^ cells), C_16_-sphingosine (LCB 16:1;2, 3.4 pmol/10^6^ cells), C_18_-3-ketosphingosine (LCB 18:1;2(keto), 2.3 pmol/10^6^ cells) and C_20_-sphingosine (LCB 20:1;2, 0.21 pmol/10^6^ cells). Notably, the ratio of C_18_-sphinganine and C_18_-sphingosine (LCB 18:1;2) levels is 10-fold and as such also agrees well with previous studies [10,11]. To our knowledge this is the first time that C_18_-3-ketosphingosine has been detected in lipid extract of mammalian cells. Based on the successful profiling of LCB species in HeLa cells we conclude that the mass-tag strategy and complementary PRM assay is a sensitive and reproducible approach for monitoring LCB molecules sample matrices of mammalian cells.

## Conclusions

Here we described a new routine for quantitative profiling of LCB species in yeast and mammalian cells. The methodology is based on chemical derivatization of LCB species using deuterated methyliodide (CD_3_I) to produce LCB analytes having a 9 Da mass-tag and a permanently positively charged quaternary amine group that improves both ionization efficiency and eliminates unwanted in-source fragmentation associated with analysis of underivatized LCB molecules. This CD_3_I-based derivatization strategy enables sensitive, specific and quantitative profiling of LCB species by applying a dedicated PRM assay executed using direct infusion and multiplexed TOF MS/MS analysis on a hybrid QqTOF mass spectrometer. Together the mass-tag strategy and the complementary PRM assay enabled detection and quantification of LCB species in lipid extracts of yeast and mammalian cells without conventional up-front LC-based separation. Notably, the methodology features a linear dynamic quantification range of three orders of magnitude and a limit of detection in the sub-pmol range within complex sample matrices. Moreover, structural characterization of derivatized 3-ketosphinganine revealed two structure-specific fragment ions that could be utilized for quantification and discrimination from typically more abundant isomeric sphingosine and monounsaturated sphinganine molecules. To our knowledge this is the first shotgun lipidomics method supporting sensitive, specific and quantitative profiling of 3-ketosphinganine species in biological sample matrices. To demonstrate the efficacy of the methodology we investigated how temperature affects the LCB composition of three *S*. *cerevisiae* strain with and without defective sphingolipid metabolism. In addition, we also showed that the methodology is applicable for monitoring the absolute abundances of LCB species in human HeLa cells.

We note that the mass-tag strategy is a generic approach that can readily be applied using other lipidomics platforms and serve as an additional routine in the repertoire of mass spectrometric acquisitions required for lipidome-wide quantification [17–19] and for functional studies of sphingolipid metabolism and its regulation. For example, the mass-tag strategy can easily be used on shotgun lipidomics platforms featuring Orbitrap mass spectrometers. Such platforms will support quantitative monitoring of CD_3_I-derivatized LCB analytes using multiplexed high resolution FTMS analysis and multi-stage fragmentation (MS^n^) [13]. Moreover, the mass-tag strategy can also be used on LC-MS-based platform which can potentially serve as an avenue for separation and quantification of isomeric 3-ketosphinganines, sphingosines and monounsaturated sphinganines. Notably, the CD_3_I-based mass-tag strategy can also be adapted for sensitive monitoring of endogenous monomethylated and dimethylated LCB species as these molecules will gain a permanent positive charge that improves their ionization and provides a specific mass offset as compared to endogenous trimethylated LCBs [4]. Finally, we note that the methodology can be employed for quantitative profiling of LCB species in other important model organisms such as *Drosophila melanogaster* and *Caenorhabditis elegans* [24].

## Supporting Information

S1 Fig Characterization of underivatized LCBs and derivatized (CD_3_)_3_-LCBs. (A) TOF MS spectrum of underivatized C_18_-sphinganine (denoted LCB 18:0;2). Protonated LCB 18:0;2 ([M+H]^+^) is isomeric with ammoniated fatty acid 18:0 (stearic acid) ([M+NH_4_]^+^). Moreover, protonated LCB 18:0;2 undergoes in-source fragmentation by neutral loss of H_2_O ([M+H-H_2_O]^+^, *m/z* 284.30). (B) TOF MS spectrum of C_18_-4-hydroxysphinganine (denoted LCB 18:0;3). Protonated LCB 18:0;3 ([M+H]^+^) undergoes in-source fragmentation by loss of H_2_O (M+H-H_2_O]^+^), *m/z* 300.29) and 2H_2_O (M+H-H_2_O]^+^), *m/z* 282.28). (C) TOF MS spectrum of CD_3_I-derivatized C_18_-sphinganine (denoted (CD_3_)_3_-LCB 18:0;2). The (CD_3_)_3_-LCB 18:0;2 ion does not undergo in-source fragmentation as evidenced by no detection of H_2_O loss ([(CD_3_)_3_-LCB 18:0;2-H_2_O]^+^) and also no detection of characteristic deuterated trimethylaminium fragment ion at *m/z* 69.14. (D) TOF MS spectrum of CD_3_I-derivatized C_18_-4-hydroxysphinganine (denoted (CD_3_)_3_-LCB 18:0;3). The (CD_3_)_3_-LCB 18:0;2 ion does not undergo in-source fragmentation as evidenced by no detection of H_2_O loss ([(CD_3_)_3_-LCB 18:0;3-H_2_O]^+^) and also no detection of characteristic deuterated trimethylaminium fragment ion at *m/z* 69.14.
(DOCX)

S2 Fig CD_3_I-derviatization improves ionization of LCB analytes. TOF MS spectrum of an equimolar mixture of CD_3_I-derivatized C_18_-sphingosine (denoted (CD_3_)_3_-LCB 18:1;2 (*m/z* 351.41, intensity = 654 counts) and underivatized C_18_-sphingosine (denoted LCB 18:1;2 (*m/z* 300.30, intensity = 68 counts) and LCB 18:1;2-H_2_O (*m/z* 282.29, intensity = 121 counts)). The intensity of (CD_3_)_3_-LCB 18:1;2 is 3.5-fold higher than that the sum of LCB 18:1;2 and its in-source fragment ion LCB 18:1;2-H_2_O).
(DOCX)

S3 Fig Structural characterization of CD3I-derivatized LCB species. (A) TOF MS/MS spectrum of derivatized C_17_-sphingosine ((CD_3_)_3_-LCB 17:1;2) using collision energy at 30 eV. (B) TOF MS/MS spectrum of derivatized C_17_-sphinganine ((CD_3_)_3_-LCB 17:1;2) using collision energy at 35 eV. The TOF MS/MS spectra were acquired using ion enhancement at *m/z* 69.14.
(DOCX)

S4 Fig Putative chemical structures of fragmentation ions released from CD_3_I- and CH_3_I-derivatized C_18_-3-ketosphinganine. (A) TOF MS/MS spectrum of CD_3_I-derivatized C_18_-3-ketosphinganine (denoted (CD_3_)_3_-LCB 18:1;2(keto)). (B) Putative structures of fragment ions released from (CD_3_)_3_-LCB 18:1;2(keto). (C) TOF MS/MS spectrum of CH_3_I-derivatized C_18_-3-ketosphinganine (denoted (CH_3_)_3_-LCB 18:1;2(keto)). (D) Putative structures of fragment ions released from (CH_3_)_3_-LCB 18:1;2(keto).
(DOCX)

S5 Fig Evaluation of CD_3_I-labeling efficiency. Cell lysates of *S*. *cerevisiae elo3*Δ were spiked with equimolar amounts of synthetic C_17_-sphingosine (denoted LCB 17:1;2) and trimethyl-C_18_-sphingosine (denoted TMLCB 18:1;2). Samples were subjected to lipid extraction, followed by CD_3_I-derivatization and PRM analysis. (A) Representative TOF MS/MS spectrum of (CD_3_)_3_-LCB 17:1;2. The intensity of *m/z* 69.14 is 28019 counts. (B) TOF MS/MS spectrum of TMLCB 18:1;2. The intensity of *m/z* 60.08 is 27411 counts). The CD_3_-labeling efficiency was estimated to be 98%±12% (n = 4) by calculating the intensity ratio of (CD_3_)_3_-LCB 17:1;2 monitored by *m/z* 69.14 (A) and TMLCB 18:1;2 monitored by *m/z* 60.08 (B).
(DOCX)

S1 Table Instrumental parameters for parallel reaction monitoring (PRM) of CD_3_I-derivatized LCB species. (PDF)

S2 Table LCB levels in yeast, pmol LCB species per OD, related to Fig 5. (XLSX)

S3 Table LCB levels in HeLa cells, pmol LCB species per 1e6 HeLa cells, related to Fig 6. (XLSX)
