# Supplementary figures and images for: Quantitative Profiling of Long-Chain Bases by Mass Tagging and Parallel Reaction Monitoring
Source: PLoS One. 2015 Dec 11;10(12):e0144817. doi: 10.1371/journal.pone.0144817 (PMC4684364; doi:10.1371/journal.pone.0144817)

| 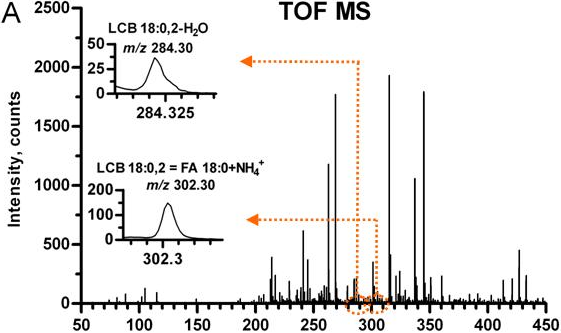 | 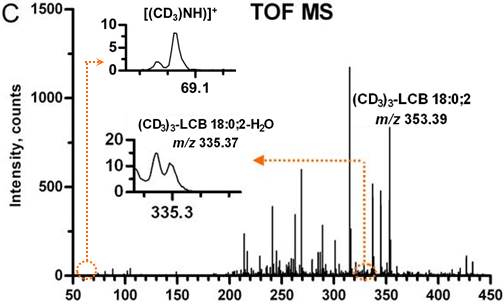 |
| --- | --- |
| 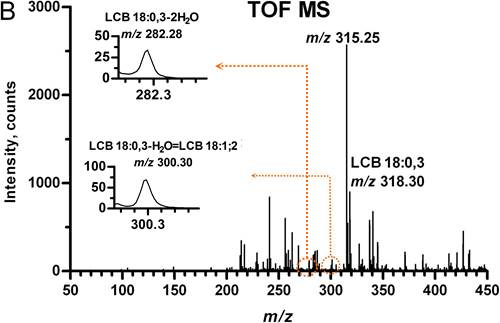 | 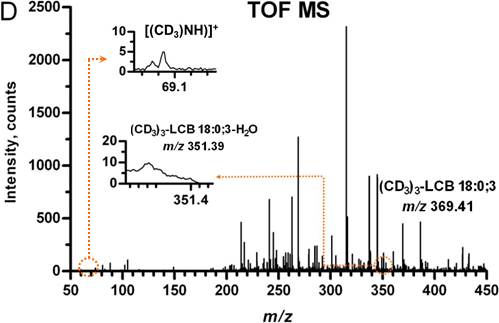 |

S1 Figure. Characterization of underivatized LCBs and derivatized (CD3)3-LCBs.

Supplement: S1 Fig — (A) TOF MS spectrum of underivatized C18-sphinganine (denoted LCB 18:0;2). Protonated LCB 18:0;2 ([M+H]+) is isomeric with ammoniated fatty acid 18:0 (stearic acid) ([M+NH4]+). Moreover, protonated LCB 18:0;2 undergoes in-source fragmentation by neutral loss of H2O ([M+H-H2O]+, m/z 284.30). (B) TOF MS spectrum of C18-4-hydroxysphinganine (denoted LCB 18:0;3). Protonated LCB 18:0;3 ([M+H]+) undergoes in-source fragmentation by loss of H2O (M+H-H2O]+), m/z 300.29) and 2H2O (M+H-H2O]+), m/z 282.28). (C) TOF MS spectrum of CD3I-derivatized C18-sphinganine (denoted (CD3)3-LCB 18:0;2). The (CD3)3-LCB 18:0;2 ion does not undergo in-source fragmentation as evidenced by no detection of H2O loss ([(CD3)3-LCB 18:0;2-H2O]+) and also no detection of characteristic deuterated trimethylaminium fragment ion at m/z 69.14. (D) TOF MS spectrum of CD3I-derivatized C18-4-hydroxysphinganine (denoted (CD3)3-LCB 18:0;3). The (CD3)3-LCB 18:0;2 ion does not undergo in-source fragmentation as evidenced by no detection of H2O loss ([(CD3)3-LCB 18:0;3-H2O]+) and also no detection of characteristic deuterated trimethylaminium fragment ion at m/z 69.14. (DOCX) [file pone.0144817.s001.docx]

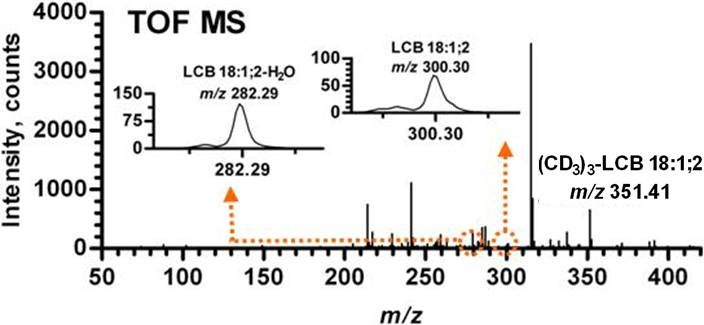


**S2 Figure. CD3I-derviatization improves ionization of LCB analytes.**

Supplement: S2 Fig — TOF MS spectrum of an equimolar mixture of CD3I-derivatized C18-sphingosine (denoted (CD3)3-LCB 18:1;2 (m/z 351.41, intensity = 654 counts) and underivatized C18-sphingosine (denoted LCB 18:1;2 (m/z 300.30, intensity = 68 counts) and LCB 18:1;2-H2O (m/z 282.29, intensity = 121 counts)). The intensity of (CD3)3-LCB 18:1;2 is 3.5-fold higher than that the sum of LCB 18:1;2 and its in-source fragment ion LCB 18:1;2-H2O). (DOCX) [file pone.0144817.s002.docx]

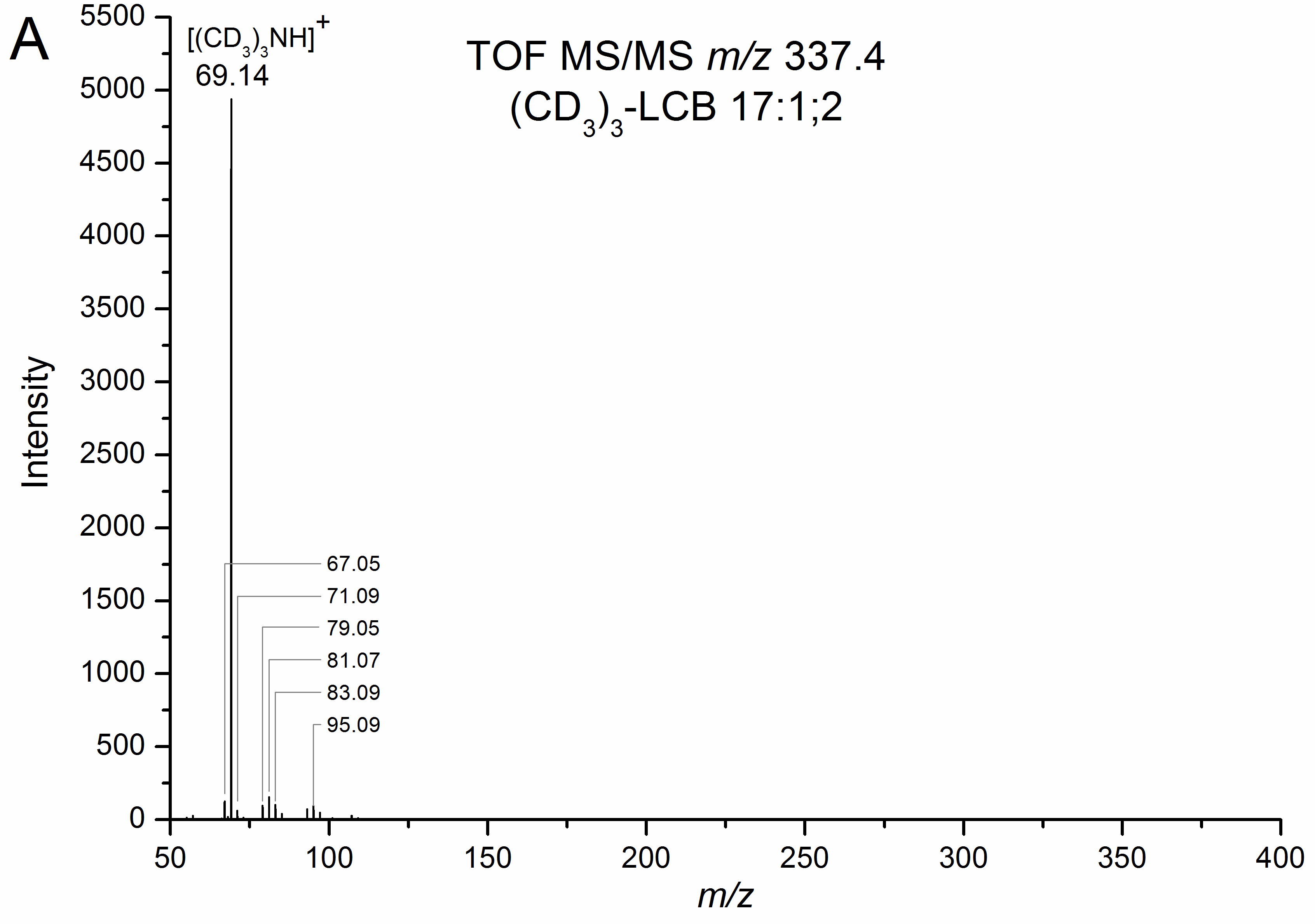

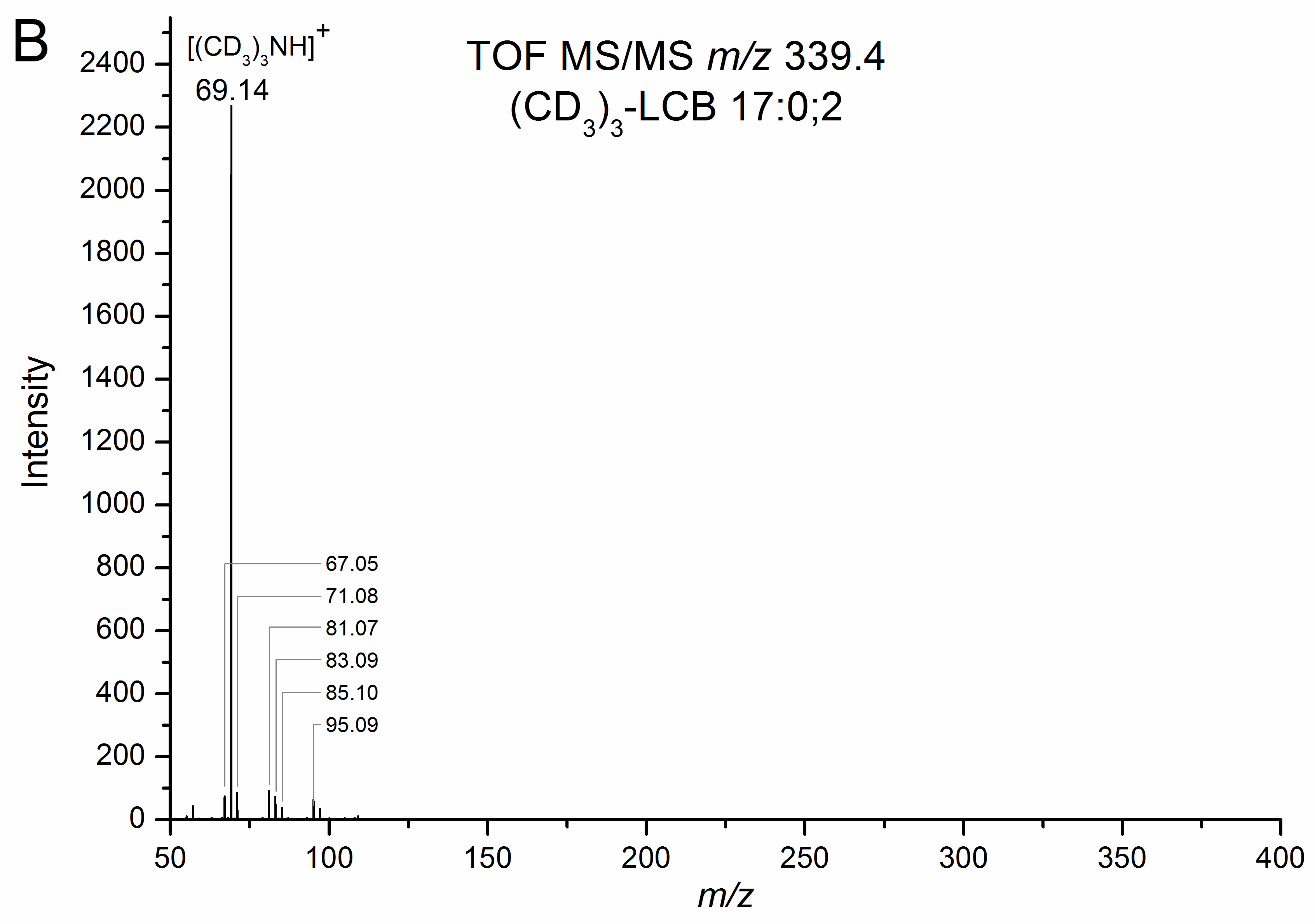


**S3 Figure. Structural characterization of CD3I-derivatized LCB species.**

Supplement: S3 Fig — (A) TOF MS/MS spectrum of derivatized C17-sphingosine ((CD3)3-LCB 17:1;2) using collision energy at 30 eV. (B) TOF MS/MS spectrum of derivatized C17-sphinganine ((CD3)3-LCB 17:1;2) using collision energy at 35 eV. The TOF MS/MS spectra were acquired using ion enhancement at m/z 69.14. (DOCX) [file pone.0144817.s003.docx]

| 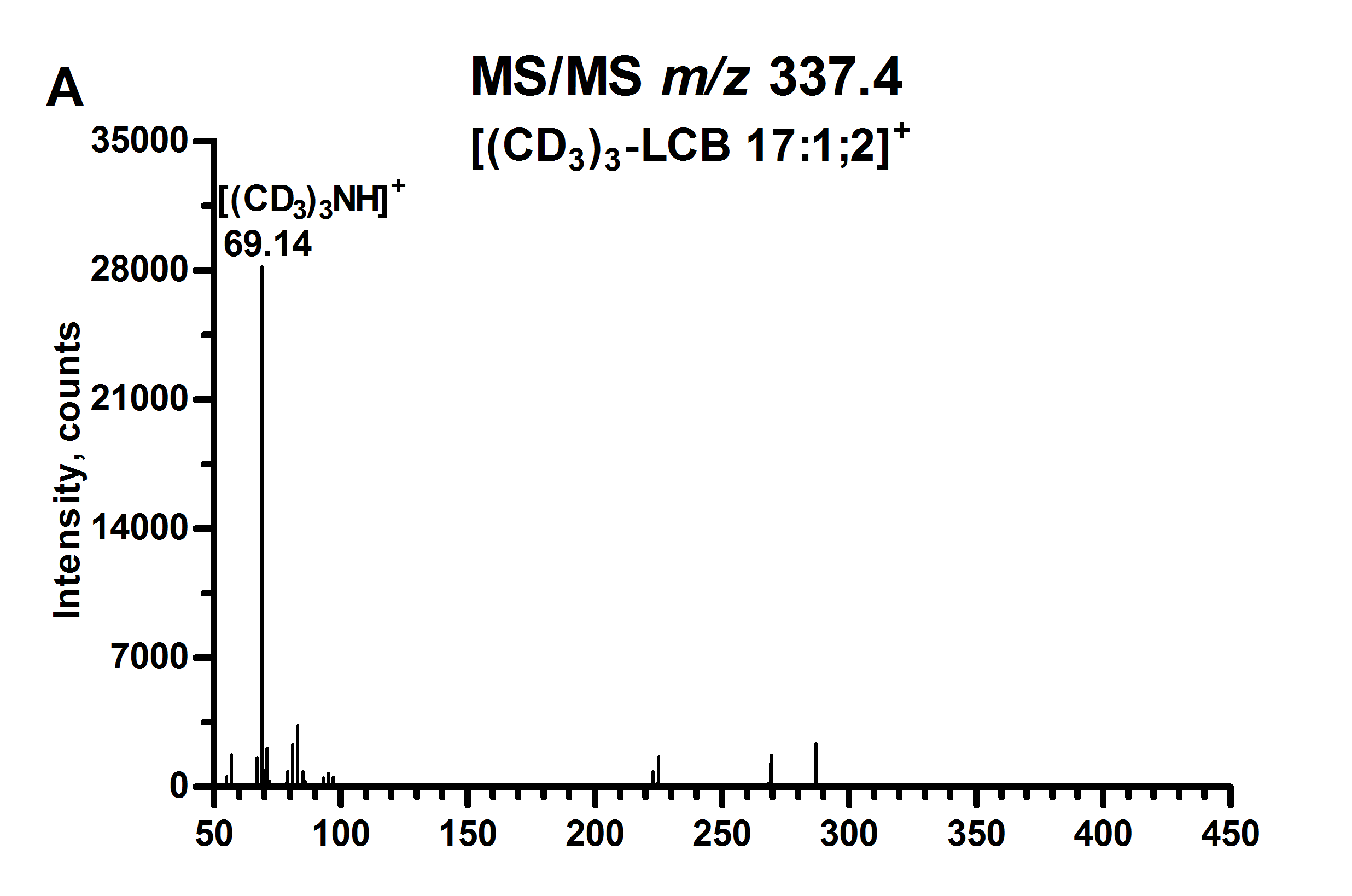 |  |
| --- | --- |
| 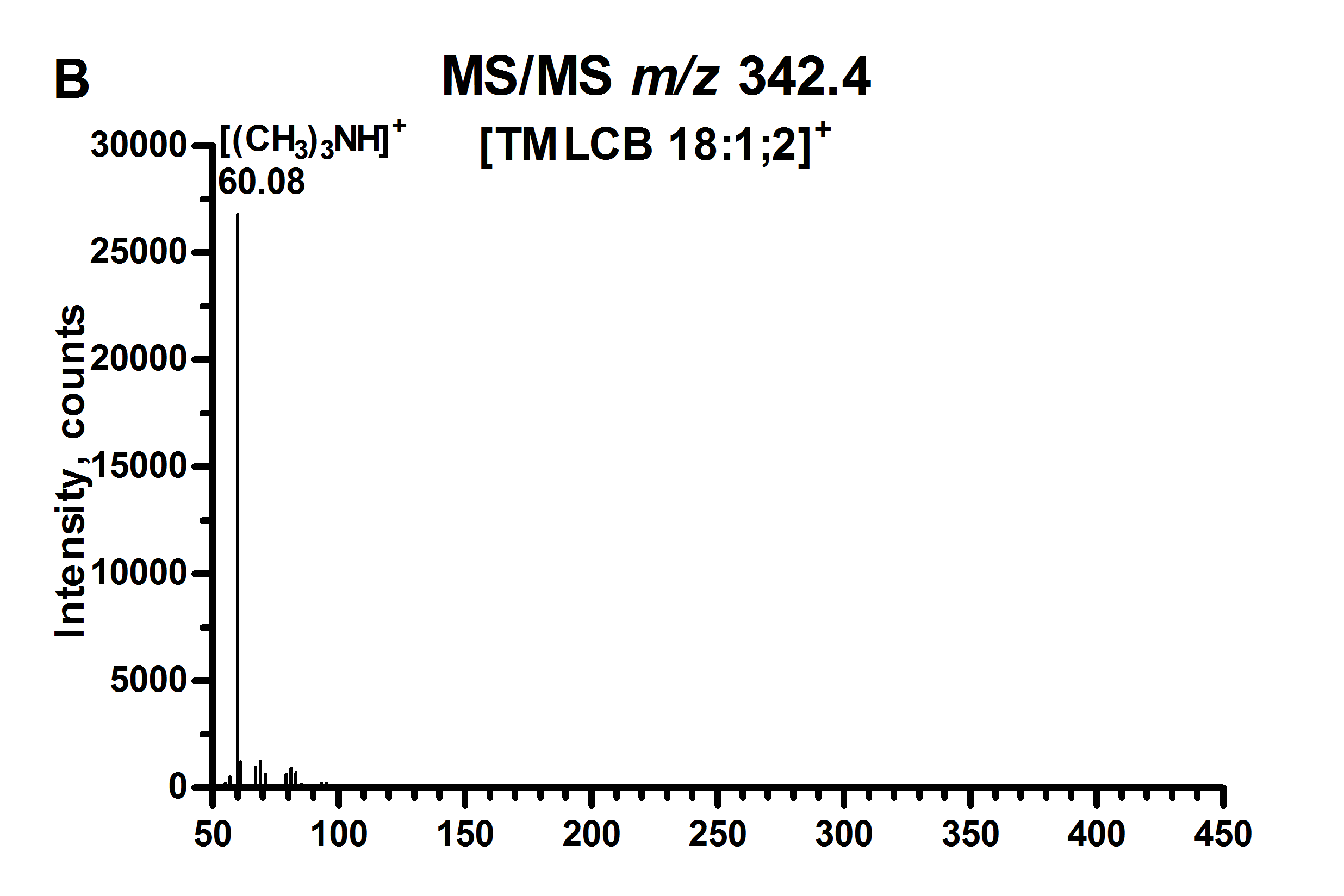 |  |
| **S5 Figure. Evaluation of CD3-labeling efficiency.** | |

Supplement: S5 Fig — Cell lysates of S. cerevisiae elo3Δ were spiked with equimolar amounts of synthetic C17-sphingosine (denoted LCB 17:1;2) and trimethyl-C18-sphingosine (denoted TMLCB 18:1;2). Samples were subjected to lipid extraction, followed by CD3I-derivatization and PRM analysis. (A) Representative TOF MS/MS spectrum of (CD3)3-LCB 17:1;2. The intensity of m/z 69.14 is 28019 counts. (B) TOF MS/MS spectrum of TMLCB 18:1;2. The intensity of m/z 60.08 is 27411 counts). The CD3-labeling efficiency was estimated to be 98%±12% (n = 4) by calculating the intensity ratio of (CD3)3-LCB 17:1;2 monitored by m/z 69.14 (A) and TMLCB 18:1;2 monitored by m/z 60.08 (B). (DOCX) [file pone.0144817.s005.docx]
