# Supplementary material for: Quantitative Profiling of Long-Chain Bases by Mass Tagging and Parallel Reaction Monitoring
Source: PLoS One. 2015 Dec 11;10(12):e0144817. doi: 10.1371/journal.pone.0144817 (PMC4684364; doi:10.1371/journal.pone.0144817)
Supplement: S4 Fig — (A) TOF MS/MS spectrum of CD3I-derivatized C18-3-ketosphinganine (denoted (CD3)3-LCB 18:1;2(keto)). (B) Putative structures of fragment ions released from (CD3)3-LCB 18:1;2(keto). (C) TOF MS/MS spectrum of CH3I-derivatized C18-3-ketosphinganine (denoted (CH3)3-LCB 18:1;2(keto)). (D) Putative structures of fragment ions released from (CH3)3-LCB 18:1;2(keto). (DOCX) [file pone.0144817.s004.docx]

C

A

| 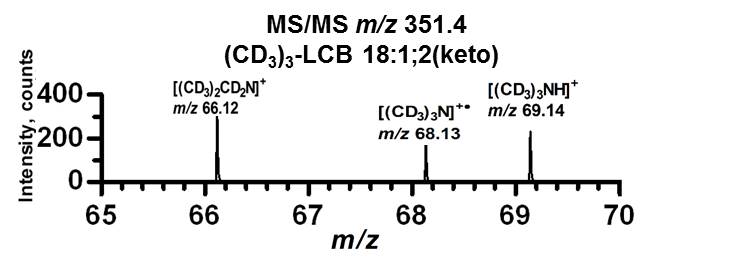  B | 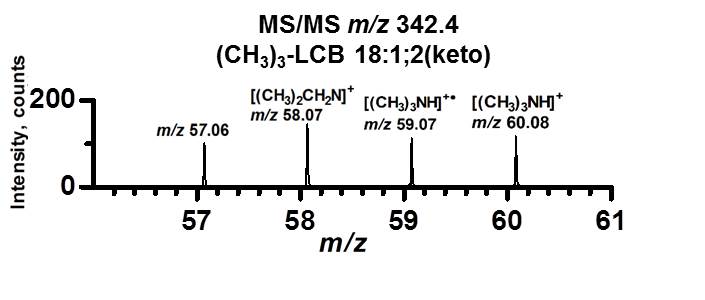  D |
| --- | --- |
| 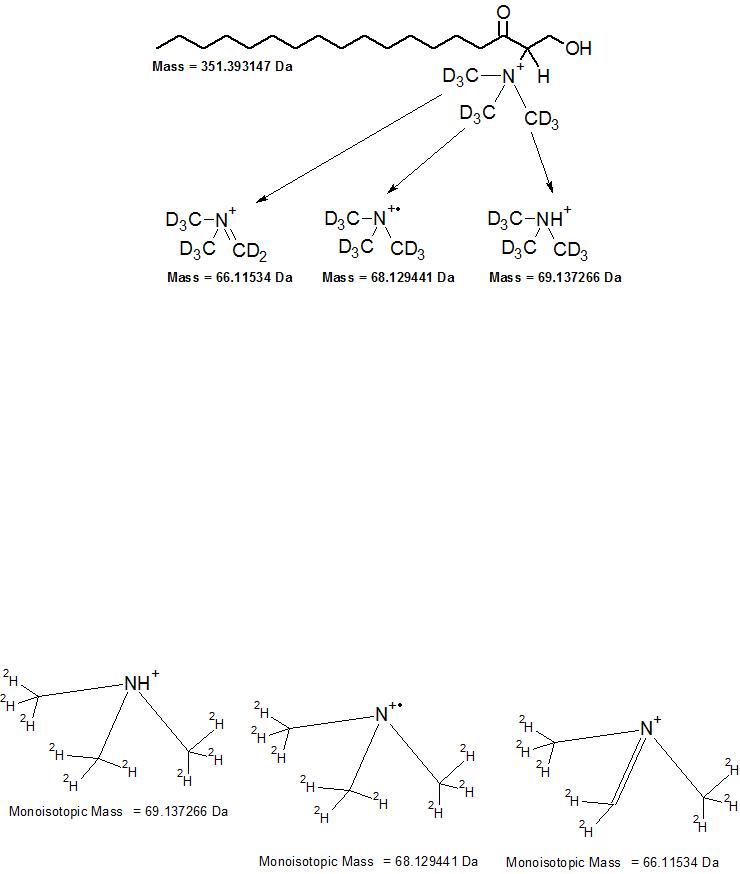 | 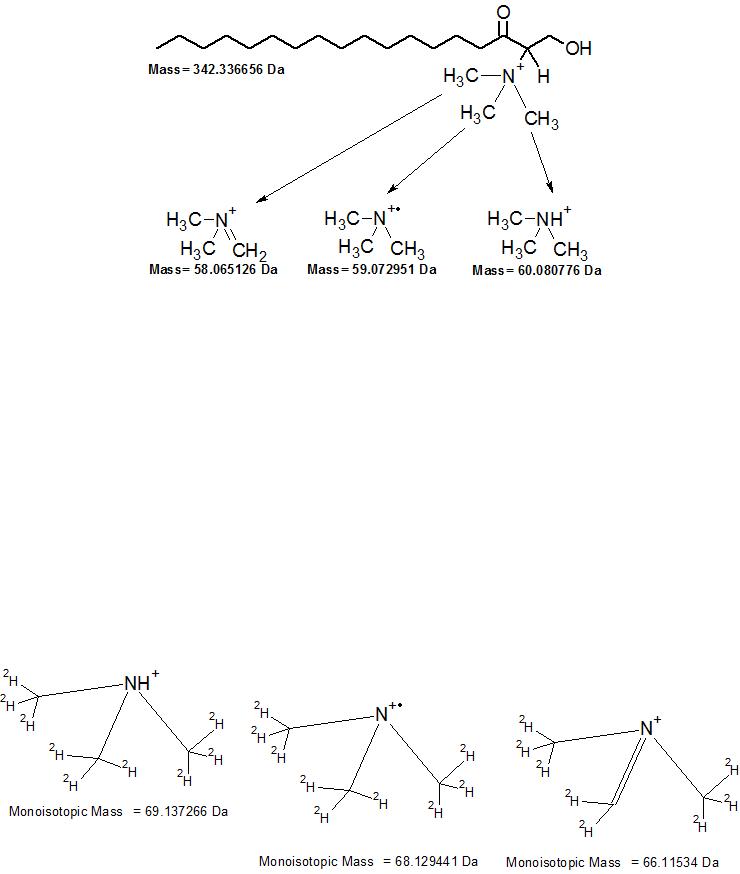 |

**S4 Figure. Putative chemical structures of fragmentation ions released from CD_3_I- and CH_3_I-derivatized C_18_-3-ketosphinganine.**
