# Supplementary material for: Quantitative Profiling of Long-Chain Bases by Mass Tagging and Parallel Reaction Monitoring
Source: PLoS One. 2015 Dec 11;10(12):e0144817. doi: 10.1371/journal.pone.0144817 (PMC4684364; doi:10.1371/journal.pone.0144817)
Supplement: S1 Table — (PDF) [file pone.0144817.s006.pdf]

S1 Table. Instrumental parameters for parallel reaction monitoring (PRM) of CD_3I-derivatized LCB species.

Lipid species	CD_3I-derivatized LCB species	MS/MS transition number	MS/MS Precursor *m/z* → Fragment ion *m/z*	Collision energy (eV)
LCB 16:1;2	$(CD_3)_3$-LCB 16:1;2	1	323.4→69.14*	30
LCB 16:1;2(keto)	$(CD_3)_3$-LCB 16:1;2(keto)	1	323.4→69.14*	30
LCB 16:1;2(keto)	$(CD_3)_3$-LCB 16:1;2(keto)	1	323.4→68.13	30
LCB 16:1;2(keto)	$(CD_3)_3$-LCB 16:1;2(keto)	1	323.4→66.12	30
LCB 16:0;2	$(CD_3)_3$-LCB 16:0;2	2	325.4→69.14	35
LCB 16:0;3	$(CD_3)_3$-LCB 16:0;3	3	341.4→69.14	35
IS LCB 17:1;2	$(CD_3)_3$-LCB 17:1;2	4	337.4→69.14	30
LCB 18:1;2	$(CD_3)_3$-LCB 18:1;2	5	351.4→69.14*	30
LCB 18:1;2(keto)	$(CD_3)_3$-LCB 18:1;2(keto)	5	351.4→69.14*	30
LCB 18:1;2(keto)	$(CD_3)_3$-LCB 18:1;2(keto)	5	351.4→68.13	30
LCB 18:1;2(keto)	$(CD_3)_3$-LCB 18:1;2(keto)	5	351.4→66.12	30
LCB 18:0;2	$(CD_3)_3$-LCB 18:0;2	6	353.4→69.14	35
LCB 18:0;3	$(CD_3)_3$-LCB 18:0;3	7	369.4→69.14	35
LCB 20:1;2	$(CD_3)_3$-LCB 20:1;2	8	379.4→69.14*	35
LCB 20:1;2(keto)	$(CD_3)_3$-LCB 20:1;2(keto)	8	379.4→69.14*	35
LCB 20:1;2(keto)	$(CD_3)_3$-LCB 20:1;2(keto)	8	379.4→68.13	35
LCB 20:1;2(keto)	$(CD_3)_3$-LCB 20:1;2(keto)	8	379.4→66.12	35
LCB 20:0;2	$(CD_3)_3$-LCB 20:0;2	9	381.4→69.14	37.5
LCB 20:0;3	$(CD_3)_3$-LCB 20:0;3	10	397.4→69.14	37.5

* The proportion of *m/z* 69.14 fragment ion intensity derived from isomeric LCB X:1;2 and LCB X:1;2(keto) (where X = {16,18,20}) were estimated using an algorithm featuring an experimentally determined fragment intensity ratio of 0.955 between the intensities of the fragment ions at *m/z* 69.14 and 66.12 for each relevant MS/MS transition. See the Materials and Methods section for details).
